# Effects of Home-Based Nine-Square Step Exercises for Fall Prevention in Thai Community-Dwelling Older Adults during a COVID-19 Lockdown: A Pilot Randomized Controlled Study

**DOI:** 10.3390/ijerph191710514

**Published:** 2022-08-24

**Authors:** Disatorn Dejvajara, Ranlaphat Aungkasuraphan, Piyathida Palee, Chanodom Piankusol, Wachiranun Sirikul, Penprapa Siviroj

**Affiliations:** 1 Faculty of Medicine, Chiang Mai University, Chiang Mai 50200, Thailand; 2 Department of Community Medicine, Faculty of Medicine, Chiang Mai University, Chiang Mai 50200, Thailand; 3 Center of Data Analytics and Knowledge Synthesis for Health Care, Chiang Mai University, Chiang Mai 50200, Thailand

**Keywords:** nine-square step exercise, fall prevention, community-dwelling older adults, COVID-19 lockdown, Thailand

## Abstract

The deterioration of muscle strength in aging has been associated with fall risks. During the COVID-19 pandemic, older adults were restricted from doing outdoor activities. This study aimed to investigate the effect of Nine-Square Step Exercises (NSSE) on improving physical performance and balance in older adults at risk of falling. We conducted an open-labelled, assessor-blinded, randomized controlled trial in 46 (aged 65–84 years) community-dwelling older adults. They were randomly assigned to an NSSE group (*n* = 24) instructed to perform the program for at least 45 days over 8 weeks or a control group (*n* = 22). The outcomes were measured by the Timed Up and Go Test (TUG), the Berg Balance Scale (BBS), the Five-Times-Sit-to-Stand test (FTSS), and hand grip strength during the baseline, 4th and 8th weeks in both groups. A mixed-effect linear regression model analysis was performed to estimate the independent effect of NSSE by the intention-to-treat over the 8-week period. The NSSE group showed significant weekly changes in BBS (β 0.57, 95% CI: 0.30, 0.84), TUG (β −0.44, 95% CI: −0.74, −0.14), and FTSS (β −0.52, 95% CI: −0.78, −0.25), demonstrating beneficial improvements in lower extremity and balance, whereas the control group did not demonstrate significant changes over time in any parameter.

## 1. Introduction

People worldwide are living longer. By the year 2030, it is estimated that one out of six people in the world will be aged 60 years or over. In addition, the world’s population of people aged 60 years and older will be expected to double to 2.1 billion by 2050 [1,2]. The health problems of the older adult will become of increasing concern and warrant more attention.

Falling is one of the most prevalent health problems among old adults. In 2018, 36 million older adults worldwide experienced a fall accident. Moreover, by the year 2030, the incidence of falls in older people is predicted to increase by 30% [3]. Falls do not only lead to hospitalization but are also a major cause of death among old adults [4]. In addition, older adults who have experienced falls are apprehensive and afraid of falling again, which results in a reduction in various activities. Moreover, the cost of medical care for both fatal and nonfatal falls was estimated to be approximately 50 billion USD [3,5,6].

Ninety percent of falls come from physiological causes such as decreased muscle strength of legs and arms and neurological dysfunction. The other 10% of falls come from external causes such as slips and trips [7,8]. The World Health Organization reports that people with decreased muscle strength have five times more risk of falling than healthy people [9]. The decrease in muscle strength occurs as a result of physiological decline and reduction in physical activities [10]. In addition, fall risks are associated with several factors such as cognitive frailty, malnutrition, impaired mobility, visual dysfunction, poor health-related quality of life, persistent pain, fall-risk-increasing drugs (FRIDs) (e.g., benzodiazepines), and drug-related side effects (e.g., sleepiness, dizziness) [11]. However, gait and balance disorders are the most common causes of falls in older adults [4]. As mentioned, the management of minimizing fall risks in older adults should be one of the most important public health concerns in the current aging society.

For more than two years, the COVID-19 pandemic has spread rapidly throughout the world [12]. In many countries, lockdown measures have been announced to control the pandemic and prevent infection in vulnerable populations [13,14,15], especially older adults, who are at a high risk from morbidity and mortality [16]. As a consequence, the average time staying at home increased, and 38% of older adults have more sedentary behavior [14]. This scenario prolongs the disuse of extremity muscles and worsens the physical condition [17]. Therefore, home-based exercises focusing on muscle strength and balance are potential interventions for preserving physical functions and reducing fall risk, especially exercises that strengthen lower extremity muscles that are necessary for body balance while standing, walking, and running [18,19].

The Nine-Square Step Exercise (NSSE), a Thai traditional dance by stepping in nine squares, was created by Ketusinh O. et al. [20,21]. A previous study of NSSE in adult and older adult populations showed improvement in balance by improving coordination among the head, eyes, and extremities that was represented by a higher equilibrium score and a lower visual analog scale (VAS) of balance symptoms [22].

Although there are many interventions that help decrease the risk of falling in older adults (e.g., Tai-Chi exercise, Lifestyle Integrated Functional Exercise, and multicomponent exercise programs) [23,24,25,26], most of them are center-based exercises that are not available and applicable during the pandemic. When health-promoting activities are restricted for older adults, home-based exercises are more practical interventions for preserving physical condition and reducing fall risk. There is a lack of evidence on home-based exercise in terms of preserving physical condition and reducing the risk of falling during the COVID-19 lockdown in community-dwelling older adults, and the benefit of home-based NSSE has not yet been clarified. We therefore conducted an open-labelled, assessor-blinded, randomized controlled trial in Thai community-dwelling older adults who are at risk of falling during the nationwide lockdown following the second outbreak of COVID-19 in Thailand (June to December 2021) [27]. This study aimed to investigate the efficacy of home-based NSSE compared to control groups on the changes of physical parameters that are associated with the risk of falling.

## 2. Materials and Methods

### 2.1. Study Design and Participants

We performed an open-labelled, assessor-blinded, randomized controlled trial in community-dwelling older adults who are at risk of falling. The eligible participants were identified from a cross-sectional study of the prevalence of cognitive frailty and its associated factors, conducted in July 2021 [28]. Briefly, the participants were community-dwelling older adults aged 65–84 years who lived in Khua Mung Subdistrict, Saraphi District, Chiang Mai Province, Thailand. This study excluded those who have been diagnosed with dementia, depression, end-stage kidney disease, hepatitis, cirrhosis, autoimmune diseases, cancer, acute trauma, acute illnesses, and those who took steroids. Details regarding sample size, participant recruitment, and data collection have been published elsewhere [27].

We recruited 373 participants from the aforementioned study and screened for fall risk using the Timed Up and Go (TUG) test in September 2021. A total of 106 older adults had a risk of falling as defined by a TUG score of more than 15 s and consented to participating in this study. The participants were excluded if they met any of the following criteria: (1) had a diagnosis of heart disease, chronic obstructive pulmonary disease (COPD), cancer, musculoskeletal diseases, or severe depression; (2) taking psychotropic, antiarrhythmic, or hypnotic drugs; (3) had a severe audiovisual impairment; (4) were living alone and did not have someone to look after them while they exercised; (5) were not willing to participate. Forty-two people declined to participate in this study due to their concern about contracting COVID-19 during the nationwide lockdown as a result of the second pandemic in Thailand.

### 2.2. Intervention

An eight-week home exercise program was assigned to the participants in the intervention group from 9 October to 4 December 2021. The Nine-Square Step Exercise (NSSE) is an exercise done by using a square block which is represented on a flat and smooth floor. The box is divided into nine equal-sized boxes (3 × 3) with total measurements of 90 × 90 cm, 120 × 120 cm, or 150 × 150 cm. The NSSE mainly requires lower extremity movement for performing exercise maneuvers within a square, as well as the contraction of abdominal and lower back muscle, and upper extremity movement to keep the body in a balance position. It is different from normal motions that occur in daily life. Those who perform the Nine-Step exercise will take slow and rapid steps in changing directions, consisting of backward, forward, and sideways directions. The Nine-Square Dance requires more coordination and includes many body turns. In accordance with the same principles as in other balance-improving exercises, these movements also require head rotation, body balance, and proprioception (visual, sensory, and vestibular function) to indicate the root position and keep the trunk in balance. This exercise program was conducted based on the protocol that all participants attended the NSSE program for at least 45 days over the 8-week study period (75% percent of days) and did at least 5 min or 5 rounds per session. The intervention group had to do the exercise continuously at home for 8 weeks and fill out the form every time they did the exercise. The participants in the non-exercise control group were asked to maintain their level of activity during the eight-week study period. During the 4th and 8th weeks, both groups met with the researchers to measure the physical parameters and collect the self-recorded exercise data from the intervention group. Then, the data were recorded as 4th-week (T1) and 8th-week (T2). We divided the two parts of the NSSE, the Nine-Square Step and the Nine-Square Dance that we used in this study as follows [22].

#### 2.2.1. Nine-Square Step Exercise

**Figure 1:**
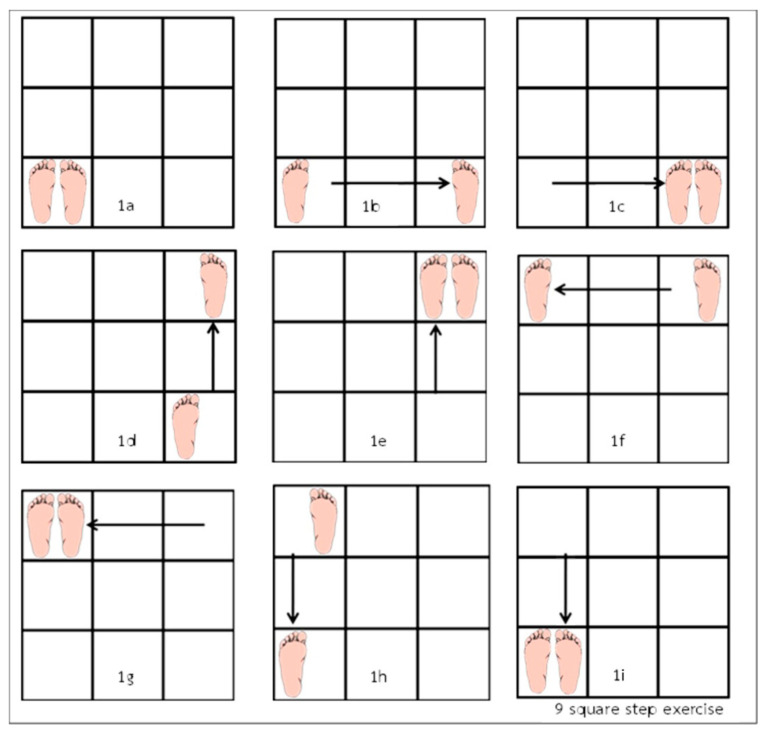
Nine-Square Step protocol [22].

(1) Preparatory position: Stand with both feet within the bottom left square (Figure 1a).
(2) Exercise steps: Moving in a counterclockwise direction, move the right foot directly to the right into the bottom right square (Figure 1b), followed then by the left foot. Now both feet are in the same square (Figure 1c). Now move the right foot straight ahead to the top right square (Figure 1d), followed by the left foot (Figure 1e). Now move the left foot directly to the left into the top left square (Figure 1f), followed by the right foot (Figure 1g). Now move the left foot directly backwards to the bottom left square, (Figure 1h), followed then by the right foot (Figure 1i), which is where you started. Now that one cycle of the exercise has been completed, move to the bottom right square to begin, except now repeat these movements going in the opposite (clockwise) direction until 5 min or 5 rounds in each side have been completed (Figure 1).

#### 2.2.2. Nine-Square Dance Exercise

**Figure 2:**
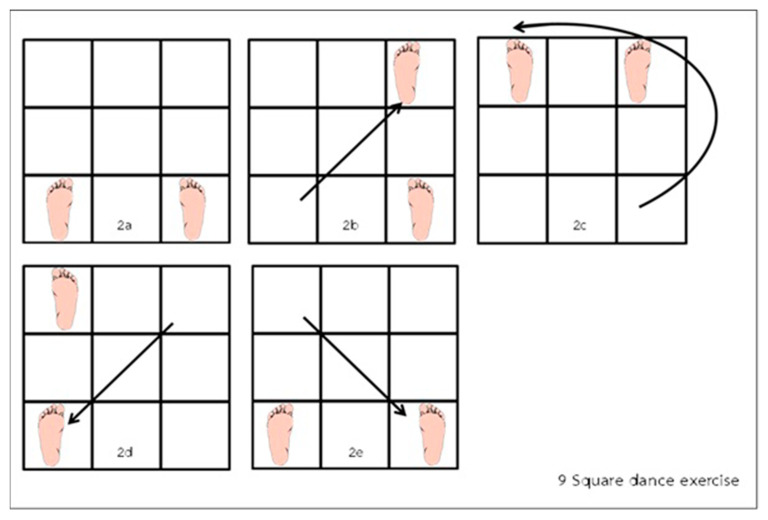
Nine-Square Dance protocol [22].

(1) Preparatory position: Stand with the feet apart, with the left foot in the bottom left square, and the right foot in the bottom right square (Figure 2a).
(2) Exercise steps: Move the left foot diagonally to the top right square (Figure 2b). Now bring the right leg around the front of your left leg and position the right foot in the top left square (Figure 2c). Now move the left foot back to the bottom left square, which is where you started with the left foot (Figure 2d), followed by movement of the right foot to the bottom right square, which is where you started with the right foot (Figure 2e). Repeat these movements going in the opposite direction for at least 7 min (Figure 2).

To reduce participant dropout and maintain adherence, we assigned village health volunteers, who have been trained by the investigators to teach the participants in the intervention group about the NSSE. Moreover, a video, poster, and exercise self-record forms were provided to the participants in the intervention group (Video S1). Other support related to usual healthcare provision for the community-dwelling older adults (e.g., periodic health screening or disease prevention) was the same in both groups in accordance with Thai standard practice for primary healthcare.

### 2.3. Measurements

The outcome was measured at the baseline (T0) and at the end of 4th (T1) and 8th (T2) week in all groups. Fall risk was evaluated by balance and leg muscle strength. The Timed Up and Go (TUG) and Berg Balance Scale (BBS) tests were used to assess balance and muscle strength. The Sit-to-Stand (STS) test was used to assess lower extremities’ muscle strength. Participants also completed a demographic questionnaire that included sex, age, BMI, smoking, alcohol use, exercise, number of underlying conditions (e.g., dementia, depression, end-stage kidney disease, hepatitis, cirrhosis, autoimmune diseases, cancer, acute trauma, acute illnesses, and taking steroids), medication use, and the Barthel Index for Activities of Daily Living (ADL). The Barthel Index for Activities of Daily Living (ADL) is a form for evaluating ten basic daily activities, including mobility, feeding, bowel and bladder control and personal care, rated by an ordinal scale with a maximum score of 20. The Thai version of the Barthel Index has intra-rater reliability (0.968), and inter-rater reliability (0.714) [29]. All of the outcomes were assessed by well-trained medical students.

### 2.4. Primary Outcomes

#### 2.4.1. Berg Balance Scale

The Berg Balance Scale (BBS) has 14 items in daily life activities and living settings to evaluate a fall risk. Each item ranges from 0 to 4. The total score is used to classify the fall risk into 3 groups: high-risk group (0–20 points), moderate-risk group (21–40 points), and low-risk group (41–56 points) [30]. A recent meta-analysis showed that BBS has a high estimated alpha coefficient (0.92, 95% CI 0.89–0.94), intra-rater reliability (0.96, 95% CI 0.94–0.97), and inter-rater reliability (0.97, 95% CI 0.95–0.98) [31].

#### 2.4.2. Timed up and Go Test

The Timed Up and Go test (TUG) was used to evaluate leg muscle strength and balance. The test starts with the participant sitting still in the upright position on a 45 cm-high chair, then has to get up and walk quickly for 3 m, turn around a barrier and return to the chair. The time is counted from the participant getting up from the chair to finishing when they return to sit on the chair. The participants who take more than 12 s to finish the walk are considered to have a high fall risk [32]. The inter rater reliability for TUG in community-dwelling older individuals is 0.96 with a 95% CI of 0.89 to 0.97 [33].

#### 2.4.3. Five-Times-Sit-to-Stand Test

The Five-Times-Sit-to-Stand test (FTSS) was used to evaluate lower extremity function. The first step of this test is sitting in an upright position on a 45 cm-high chair, then getting up and sitting down on the chair quickly 5 times without any physical assistance. The time is measured from the participant getting up from the chair to finishing when the participant sits down for the fifth time. A duration longer than 15 s is considered as a high risk of falling [34,35]. The estimated FTSS test-retest reliability from a meta-analysis is 0.94 with a 95% CI of 0.88 to 0.97 [36]. The FTSS test shows excellent intra-rater reliability (intraclass correlation coefficient (ICC) range: 0.91–0.93) and moderate intra-rater reliability (ICC range: 0.64–0.88) in healthy older and young subject groups, respectively. Inter-rater reliability between assessors yields an ICC of 0.99 [37].

#### 2.4.4. Hand Grip Strength

Hand grip strength, estimated by a digital hand dynamometer (TAKEI T.K.K.5401^®^, Takei Scientific Instruments Co., Ltd., Tokyo, Japan), was used to estimate upper body muscle strength. The test was performed by using the dominant hand to grab the tool then squeezing it with all their strength 3 times with a rest of 15–20 s between measurements. While they were squeezing, the participant must stand erect with their hand toward the floor. The time recorded was the average of the three attempts [38,39]. Hand grip strength measurements demonstrate good to excellent test-retest reliability (>0.80) [40], intra-rater reliability (0.85–0.98), and excellent inter-rater reliability (0.94–0.98) [41].

### 2.5. Sample Size Calculation

**Figure 3:**
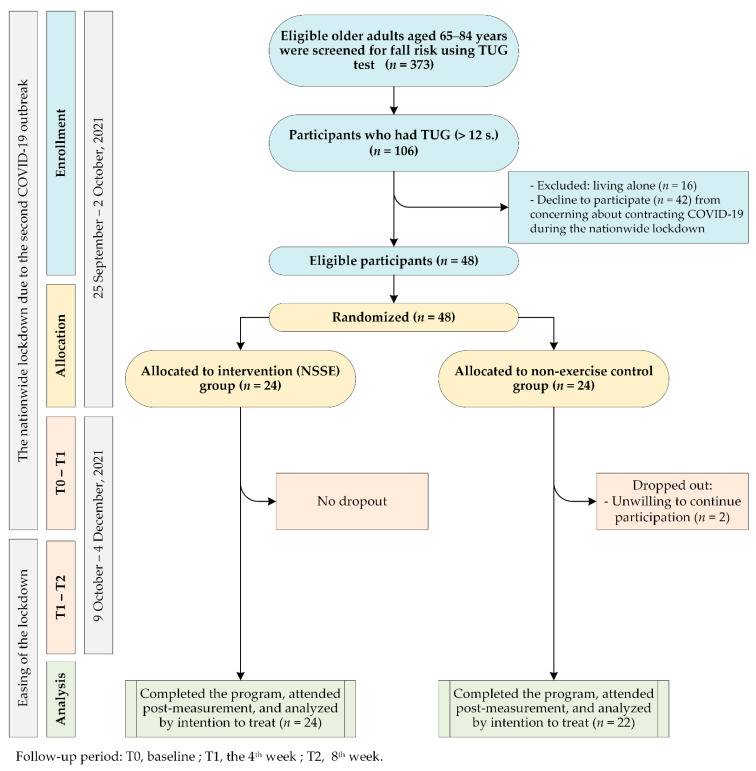
The flow diagram of the participants through each stage of the study. TUG, Timed Up and Go.

The primary outcomes were the mean differences between functional parameters at the baseline and at the end of the follow-up period, including the Berg Balance Scale (BBS), the Timed Up and Go test (TUG), the Five-Times-Sit-to-Stand test (FTSS), and hand grip strength. The expected effect size for a two-sample paired-means test was calculated using STATA 16. With 20 participants in each group, a two-tailed alpha level of 0.05, and 80% power, the expected effect size (±SD) of 0.62 (±1) could be detected. Allowing for a 20% dropout rate, forty-eight participants were included to detect the effect size from a previous calculation and then randomized into the intervention group (*n* = 24) and the control group (*n* = 24). Before the 4-week outcome measurement, two participants in the control group withdrew due to unwillingness to participate. The sample population in the final analysis was 46 participants: twenty-four (6 males and 18 females) in the intervention group and 22 (8 males, 14 females) in the control group (Figure 3).

### 2.6. Randomization and Blinding

Randomization was performed using simple randomization by a computerized random sequence generator. The letters for NSSE and non-exercise control groups were placed into sequentially numbered opaque envelopes sealed by the statistician. The allocation sequence was concealed from all investigators, and all outcome assessors were blinded to participant randomization assignment. We regularly informed participants and their caregivers not to disclose their randomization assignments to the outcome assessment team and other participants.

### 2.7. Statistical Analysis

For continuous variables, the central estimates with variabilities of data, a histogram with a density plot, and the Shapiro–Wilk test were used to assess the normality of data distribution. A mean with a standard deviation (SD) was used to describe parametric continuous variables. The median and interquartile range (IQR) were used to present nonparametric continuous variables. A frequency with a percentage was used for categorical variables. The baseline characteristics of the two groups were compared using Fisher’s exact test for categorical variables, the independent sample t-test for parametric continuous variables, and the rank sum test for nonparametric continuous variables. A mixed-effect linear regression model was performed to estimate the independent effect of NSSE on physical performance parameters, adjusted for time effects due to repeated outcome measurements. An adjusted β-coefficient with a 95% CI from the models was presented to indicate NSSE effects and time effects on non-exercise control participants. An analysis of variance (ANOVA) test with postestimation for effect size (partial eta squared, ηp^2^) was performed. All statistical analyses were performed via using statistical software STATA (Stata Corp., 2019, College Station, TX, USA, Stata Statistical Software: Release 16, Stata Corp., LLC). The *p*-value was deemed statistically significant at 0.05 (two-tailed). The study was reported in accordance with the Consolidated Standards of Reporting Trials (CONSORT) 2010 statement [42].

## 3. Results

### 3.1. Baseline Characteristics

The baseline characteristics of participants are shown in Table 1. There were no significant differences in baseline characteristics between the NSSE and the non-exercise control group in terms of demographic data or health profiles consisting of sex, age, marital status, body mass index (BMI), smoking, alcohol drinking, current exercise, underlining diseases, medications used, hand grip strength, and the Barthel Index for Activities of Daily Living (ADL). The mean (±SD) ages were 69.8 (±3.6) and 71.9 (±5.2) in the NSSE and control groups, respectively. The majority in both groups was female, married, of moderate weight or overweight, non-smoking, non-alcohol-drinking, exercised more than 3 days per week, had one or two underlying diseases, and used one medication. Screening for the mean score of physical parameters at the baseline and ADL were not significantly different between the two groups (*p* > 0.05).

### 3.2. Changes in Physical Performance during the 8-Week Intervention Period

**Figure 4:**
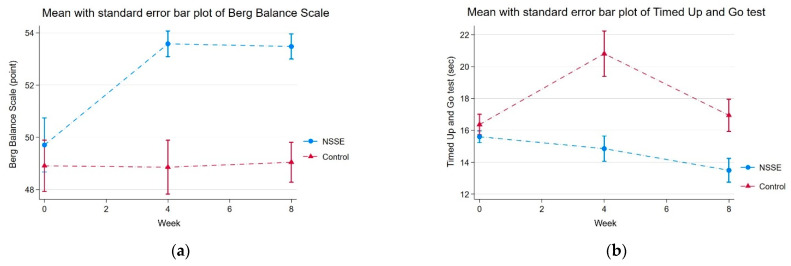

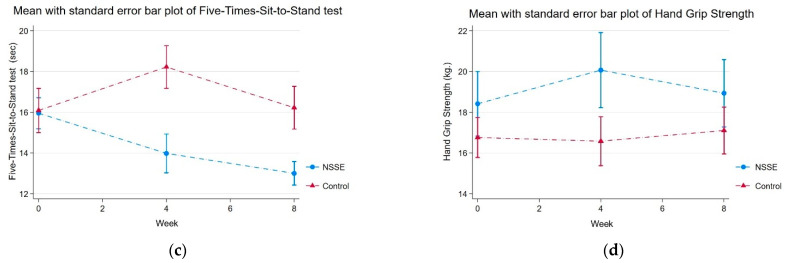
Changes in physical performances summarized by the mean with standard error bar plots. (**a**) Changes in the Timed Up and Go test; (**b**) changes in the Berg Balance Scale; (**c**) changes in Five Times Sit to Stand; (**d**) changes in hand grip strength.

Figure 4 illustrates the unadjusted changes in physical performance summarized by the group mean with standard error bar plots of NSSE and control groups at baseline (T0), 4 weeks (T1), and 8 weeks (T2). At the baseline (T0), there was no difference of physical performance parameters between the NSSE and the non-exercise control groups. Compared to the baseline data (T0), the NSSE group showed an increase in BBS (Figure 4a) over the follow-up period. Although the NSSE group showed a slight increase in hand grip strength at the 4th week, there was no difference in hand grip strength between the two groups at the 8th week. For TUG (Figure 4b) and FTSS (Figure 4c), the average times of the NSSE group decreased over the 8-week period. In contrast, the non-exercise group showed a fluctuation in the average TUG and FTSS times, which increased in the 4th week and decreased in the 8th week.

The primary outcomes of each group during 8-week follow-up period are shown in Table 2. After adjustment for time effects, the primary outcomes, including the Berg Balance Scale (BBS), Timed Up and Go (TUG), and Five-Times-Sit-to-Stand (FTSS) were significantly improved in the NSSE group compared to those in the non-exercise control group, while changes in physical performance parameters in the control group did not decline significantly over the follow-up period. The Berg Balance Scale (BBS) in the NSSE groups was significantly increased on each intervention week (β-coefficient 0.57, 95% CI 0.30 to 0.84). Changes in TUG (β-coefficient −0.44, 95% CI −0.74 to −0.14) and FTSS (β-coefficient −0.52, 95% CI −0.78 to −0.25) in the NSSE group were also significantly lower than in the non-exercise control group at each intervention week. Changes in hand grip strength were not significantly different between the NSSE and control groups at each intervention week (β-coefficient 0.03, 95% CI −0.19 to 0.30).

## 4. Discussion

Falling is a detrimental health issue among older adults. As a consequence of the COVID-19 lockdown, older adults tended to become more inactive, resulting in a deterioration in their physical condition and an increased risk of falling. Our study revealed that the home-based NSSE was beneficial in reducing the risk of falls in older adults, as indicated by significant improvements in primary outcomes (the Berg Balance Scale (BBS), Timed Up and Go (TUG), and Five-Times Sit-to-Stand (FTSS)) over the 8-week intervention period.

Balance capacity is characterized as the ability to control, maintain, or recover a state of posture in any activity [43]. The Berg Balance Scale is one of the most widely used clinical tests for assessing static and dynamic balance in older adults. An old adult is considered to be at a high risk of falling if they score less than 41 without a history of falls [34,44,45,46] or less than 51 with a history of falls [46]. Although the BBSs in both the NSSE and non-exercise control groups were greater than 41, the NSSE intervention significantly increased the BBS compared to that in the non-exercise control group over the eight-week period. Only the NSSE group showed a significantly high mean BBS, nearly the maximum BBS of 56, indicating high functional balance at the fourth and eighth weeks. From a previous study in post-stroke patients, the minimal clinically important difference (MCID) of BBS was estimated by using the Functional Ambulation Categories (FAC) as anchors for changes in BBS scores. The estimated MCID of BBS scores in the unassisted-walking group was four points [47]. In the NSSE group, we found that the mean difference of BBS between T0 and T2 was 4.04 (95% CI 2.22 to 5.86), indicating a clinically significant difference in physical function. Half of the NSSE group achieved the MCID level of BBS. In addition, we evaluated FTSS and TUG, which are widely used in research and clinical settings to assess the strength of lower extremity muscles, the progression of balance from sitting to standing and walking, and to screen individuals at increased risk of falling. The TUG and FTSS tests measured balance and lower limb strength, which consists of eight major lower limb muscles, namely hip flexors, hip extensors, hip abductors, hip adductors, knee flexors, knee extensors, ankle dorsiflexors, and ankle plantar flexors. These tests were also performed with the subject’s arms crossed across his or her chest. This position limits the contribution of the upper limbs to body balance and raises the subject’s center of mass, which makes it harder for the subject to keep their balance. Individuals with a TUG higher than 12 s are considered to be at risk of falling, and this threshold was used to include those at risk in our study [48]. The MCID levels of TUG have been variously reported in specific diseases, but there has not been a specific study on the functional improvement of community-dwelling old adults. The most relevant MCID of TUD might be 0.8 s as reported in a study of hip osteoarthritis patients anchored with the global rating of change score (GRCS) [49]. In the NSSE group, we found that the mean difference of TUG between T2 and T0 was 2.20 (95% CI 0.66 to 3.74), and 70.8% of the NSSE group had a significant change as defined by an MCID of 0.8 s. The significant decrease in TUG time of the NSSE group compared to that in the non-exercise control group also confirmed the benefit of home-based NSSE, which not only preserved balance capacity but also improved it and could reduce the risk of falls among older adults [50]. As a result of the lockdown, we also found that the non-exercise control group had increased TUG time at the fourth week and returned to the baseline at the 8th week. This fluctuation may be explained by physical activity gaining from their occupation and daily activities as the lockdown measures relaxed. The averages of TUG time and BBS in the non-exercise control group did not significantly decrease at the end of the study and over the follow-up period according to a time effect from no physical activity and the lockdown. The TUG time of these participants remained at a level associated with a risk of falling.

Additionally, our results showed that FTSS time, indicative of functional lower extremity strength [34,35], significantly decreased each week in the NSSE group but not in the non-exercise control group. The reported MCID level of FTSS was greater than 2.3 s anchored with gait speed, TUG, the Dynamic Gait Index, the Dizziness Handicap Inventory, and the Activities-Specific Balance Confidence Scale for demonstrating clinical improvement in balance and vestibular disorders [51]. In the NSSE group, the mean difference of FTSS between T2 and T0 was 2.93 (95% CI 1.35 to 4.52), and 54.2% of the NSSE group had a significant change as defined by an MCID of 2.3 s. Similar to TUG, the fluctuation of FTSS in the non-exercise control group was also observed and may be explained by the aforementioned reasons. The change of FTSS in the non-exercise control group was not significant from the mixed-effect analysis, and the average FTSS time of non-exercise control participants remained greater than 15 s at the end of the trial, which is considered a high risk of falling in older adults [52]. In this study, we also assessed hand grip strength, which is an indicator of general muscle and upper extremity strength as well as the risk of fragility and falling [53,54]. However, there was no significant change in hand grip strength between the NSSE and the non-exercise control group over the follow-up period. Since the home-based NSSE program mainly focused on balance and lower extremity strength, this may explain why NSSE failed to demonstrate a significant improvement in hand grip strength [55].

To the best of our knowledge, this was the first open-labeled, randomized controlled study assessing the benefits of a home-based exercise program on the physical performance of community-dwelling older adults during a COVID-19 lockdown. Major strengths of this study include the randomized experimental design and the mixed-effect regression analysis used to examine the independent effects of NSSE. Although the sample size was relatively small, we ensured that our study had sufficient statistical power to detect the effect of NSSE on the primary outcomes as declared in Section 2.5. Nevertheless, there were several limitations to this study that need to be clarified. First, the estimate of NSSE and time effects during the lockdown period should be interpreted with caution due to the improvement in the pandemic situation after the 4th week of the study period. As a result of the easing of lockdown restrictions, some participants in both groups might have engaged in greater physical activity than they did during the lockdown period, as they resumed their normal daily routines and returned to work. Thus, the effect of the lockdown restrictions on physical deterioration may have been underestimated and the effects of NSSE may have been overestimated. Second, the eight-week study period is relatively short. Our study was unable to directly ascertain the incidence of falls but assessed the surrogate outcomes of fall risk. Although improving balance capacity and lower extremity strength may indicate a reduction in fall risk, a study with a longer follow-up time is still necessary to determine the effectiveness of home-based NSSE for fall prevention. Third, a single community-based study may not be relevant to other communities in different social contexts. The effectiveness of home-based NSSE for fall prevention in various settings should be further investigated. Fourth, we did not assess gait ability evaluation and gait motion analysis, which provide another perspective of the patient’s condition for fall risk assessment. However, the data collection requires the experience of domain-expertise physicians or therapists to ensure that the collected data are standardized and correct. These operator-dependent evaluations may not be feasible in a community-based study. Additionally, a previous study that compared the clinical performance of the TUG test and gait assessment found that the TUG test is more useful for the clinical assessment of mobility and the risk of falling than gait assessment [56]. Lastly, to mitigate the risk of COVID-19 infection during the study period, intra- and inter-rater reliabilities of these instruments were not assessed because one assessor was assigned to investigate the outcome of each patient without re-examination by other assessors.

The results from this pilot study showed a beneficial improvement in physical strength and balance, which could help reduce fall risk within a short period of time. In a previous study, the NSSE also showed the benefit of improving symptoms and balance in patients with balance disorders after 8 weeks of exercise when evaluated by a visual analogue scale and computerized dynamic posturography [22]. In comparison to other balance exercise, this exercise is safe and easy to perform at home without specific equipment. It has been recommended as an exercise technique for improving cardiovascular and physical fitness in community-dwelling older adults in Thailand for more than 10 years. Moreover, this exercise can be done sustainably due to the fact that village health volunteers and family members were knowledge providers in this experiment. This home-based intervention can be incorporated into primary health care as a health promotion service to reduce fall risk among the community-dwelling older adults. Furthermore, the study of NSSE combined with upper body exercise is encouraged to investigate an additional benefit on upper extremity strength and general physical condition. For a future study, we plan to integrate motion capture technology (Depth camera technology) [57] with NSSE to implement a digital health intervention [58] for older adults and a return-to-work assessment (e.g., post-stroke, post-injury patients). In this project, we aim to develop and validate the digital health intervention for assessing physical performance and frailty risk and providing an interactive home-based exercise based on NSSE. The NSSE movement analysis from the Depth camera can identify abnormalities or individuals at risk for frailty by a slow reaction time and the degree of joint flexibility from the estimation of motion points. As a digital health intervention, it can provide several potential advantages, including continuous monitoring of compliance and physical performance data and more personalized exercises based on the patient’s condition. We also encourage researchers in this area to investigate the incorporation of digital technologies and culturally specific exercises for physical function improvement and fall prevention in their particular contexts.

## 5. Conclusions

The home-based Nine-Square Step Exercise (NSSE) demonstrated beneficial improvements in lower extremity strength and balance, which could help reduce fall risk in a short period of time. With the assistance of healthcare nonprofessionals—family members or health volunteers—this exercise can be performed sustainably without any equipment or complicated procedures. In order to determine the effectiveness of home-based NSSE for fall prevention, a study with a longer follow-up period in other settings is still required.

## Figures and Tables

**Table 1:** Baseline descriptive data of the participants.

Characteristics	NSSE Group (*n* = 24), *n* (%)	Non-Exercise Control Group (*n* = 22), *n* (%)	*p*-Value
Sex			
Male	6 (25.0)	8 (36.4)	0.525 ^a^
Female	18 (75.0)	14 (63.6)	
Age (years), mean ± SD	69.8 ± 3.6	71.9 ± 5.2	0.118 ^d^
65–69	13 (54.2)	9 (40.9)	0.168 ^b^
70–74	9 (37.5)	6 (27.3)	
≥75	2 (8.3)	7 (31.8)	
Marital status			
Single	2 (8.3)	4 (18.2)	0.545 ^b^
Married	15 (62.5)	11 (50.0)	
Divorced	7 (29.2)	7 (31.8)	
BMI (kg/m^2^), mean ± SD	22.44 ± 4.12	23.50 ± 4.94	0.435 ^c^
Underweight < 18.5	3 (12.5)	2 (9.1)	0.696 ^b^
Normal 18.5–22.9	10 (41.7)	7 (31.8)	
Overweight 23.0–27.5	11 (45.8)	13 (59.1)	
Smoking			
Never	16 (66.7)	15 (68.2)	0.801 ^b^
Former	7 (29.1)	5 (22.7)	
Current	1 (4.2)	2 (9.1)	
Alcohol drinking			
Never	17 (70.8)	15 (68.2)	0.306 ^b^
Former	6 (25.0)	3 (13.6)	
Current	1 (4.2)	4 (18.2)	
Current exercise			
No	3 (12.5)	2 (9.1)	0.913 ^b^
1–2 days/week	8 (33.3)	6 (27.3)	
3 days or more/week	13 (54.2)	14 (63.6)	
Number of underlying diseases, median (IQR)	1 (1–2)	2 (1–2)	0.069 ^c^
Number of medications used, median (IQR)	1 (1–2)	1 (1–2)	0.298 ^c^
Berg Balance Scale, mean ± SD	49.7 ± 1.0	48.9 ± 1.0	0.580 ^d^
Timed Up and Go test (s), mean ± SD	15.6 ± 0.4	16.4 ± 0.6	0.299 ^d^
Five-Times-Sit-to-Stand test (s), mean ± SD	15.9 ± 0.8	16.1 ± 1.1	0.918 ^d^
Hand grip strength (kg), mean ± SD	18.4 ± 1.6	16.8 ± 1.0	0.389 ^d^
Barthel Index for Activities of Daily Living (ADL), median (IQR)	20 (19.0–20.0)	20.0 (18.7–20.0)	0.300 ^c^

^a^ Analyzed by chi-square test, ^b^ analyzed by Fisher’s exact test, ^c^ Mann–Whitney U test, ^d^ independent sample *t*-test; NSSE, Nine-Square Step Exercise; BMI, body mass index; SD, standard deviation; Q, quartiles.

**Table 2:** Changes in physical performance parameters in the NSSE group and the control group over the 8-week period.

Primary Outcomes	Study Groups	Adjusted β-Co-Efficient (Change per Week)	95% CI	*p*-Value	ηp^2^	95% CI
Berg Balance Scale (BBS)	NSSE	0.57	0.30 to 0.84	<0.001	0.14	0.05 to 0.25
	Control	−0.35	−0.25 to 0.18	0.747	0.05	<0.01 to 0.13
Timed Up and Go (TUG)	NSSE	−0.44	−0.74 to −0.14	0.004	0.14	0.04 to 0.25
	Control	0.11	−0.11 to 0.35	0.337	0.07	0.01 to 0.17
Five Times Sit to Stand (FTSS)	NSSE	−0.52	−0.78 to −0.25	<0.001	0.08	0.01 to 0.18
	Control	0.09	−0.13 to 0.31	0.424	0.03	<0.01 to 0.10
Hand grip strength	NSSE	0.05	−0.19 to 0.30	0.673	0.03	<0.01 to 0.11
	Control	0.03	−0.14 to 0.21	0.703	<0.01	<0.01 to 0.02

Adjusted β-coefficient obtained from a mixed-effect regression model adjusted for time effects due to repeated outcome measurements. Adjusted β-coefficient of the non-exercise control group represented the changes in the parameters due to the time effects of the lockdown. Partial eta squared obtained from an analysis of variance (ANOVA) test with postestimation for effect size: categorized as small (ηp^2^ = 0.01), medium (ηp^2^ = 0.06), and large (ηp^2^ = 0.14). Control, non-exercise control group; ηp^2^**;** partial eta squared; NSSE, Nine-Square Step Exercise group; 95% CI, 95% confidence interval.

## Data Availability

The data presented in this study are available on request from the correspondent author.

## Supplementary Materials

The following supporting information can be downloaded at: https://www.mdpi.com/article/10.3390/ijerph191710514/s1, Video S1: The NSSE protocol, Nine-Square Step and Nine-Square Dance.
